# Transcatheter Aortic Valve Implantation

**DOI:** 10.2174/1573403X09666131202120413

**Published:** 2013-11

Markus Krane, Marcus-André Deutsch, Sabine Bleiziffer and Rüdiger Lange

During the past decades, the only effective treatment option for severe, symptomatic, aortic valve stenosis was surgical aortic valve replacement. More than 10 years ago, Alain Cribier performed the first transcatheter aortic valve implantation (TAVI). Since 2007, CE-mark certified aortic valves are available for TAVI. This new technology rapidly achieved a very high clinical acceptance as a treatment option, in patients considered to be at high risk for surgical aortic valve replacement. Today, more than 80.000 patients in more than 40 countries have undergone TAVI. Despite a very-high-risk patient profile, data from multi- and single-center registries have confirmed the safety and efficacy of the procedure. The randomized, controlled PARTNER trial has confirmed both a superiority of TAVI over medical treatment in patients deemed ineligible for conventional surgical aortic valve replacement and a noninferiority of TAVI in comparison to surgical aortic valve replacement in high-risk patients. Although residual, mostly trivial or mild, paravalvular aortic regurgitation is frequent, promising preliminary data exist for the long-term outcome following TAVI. Cerebrovascular and vascular complications as well as atrioventricular block leading to permanent pacemaker implantation remain the most relevant periprocedural complications after TAVI. Next generation heart valves addressing some of these issues are currently under evaluation. In the future, valve-in-valve procedures for the treatment of a degenerated bioprosthesis will become an increasingly important application of transcatheter heart valves.

The Mini Hot Topic issue “Transcatheter Aortic Valve Implantation” summarizes the current knowledge about technical aspects and treatment efficiency of TAVI in patients with severe symptomatic, aortic valve stenosis. Elhmidi *et al.* review the recently published data reporting on survival, durability and hemodynamic performance of TAVI. Bleiziffer *et al.* discuss the different access sites for TAVI including possible advantages and limitations. Moreover, Blumenstein *et al.* provide an overview about currently commercially available transcatheter heart valves and novel devices that have entered initial clinical trials more recently. Finally, Deutsch *et al.* summarize and discuss the effect of TAVI on health-related quality of life.

Our growing knowledge, the introduction of next generation devices along with a confirmed long-term durability of transcatheter heart valve prostheses will pave the way for the expansion of TAVI.

